# Drug Candidate BGP-15 Prevents Isoproterenol-Induced Arrhythmias and Alters Heart Rate Variability (HRV) in Telemetry-Implanted Rats

**DOI:** 10.3390/ph16030359

**Published:** 2023-02-26

**Authors:** Brigitta Bernat, Rita Erdelyi, Laszlo Fazekas, Greta Garami, Reka Maria Szekeres, Barbara Takacs, Mariann Bombicz, Balazs Varga, Fruzsina Sarkany, Arnold Peter Raduly, Dana Diana Romanescu, Zoltan Papp, Attila Toth, Zoltan Szilvassy, Bela Juhasz, Daniel Priksz

**Affiliations:** 1 Department of Pharmacology and Pharmacotherapy, Faculty of Medicine, University of Debrecen, H-4032 Debrecen, Hungary; 2 Department of Dentoalveolar Surgery, Faculty of Dentistry, University of Debrecen, H-4032 Debrecen, Hungary; 3 Department of Operative Techniques and Surgical Research, Faculty of Medicine, University of Debrecen, H-4032 Debrecen, Hungary; 4 Division of Clinical Physiology, Department of Cardiology, Faculty of Medicine, University of Debrecen, H-4032 Debrecen, Hungary; 5 Department of Diabetology, Pelican Clinical Hospital, Faculty of Medicine and Pharmacy, University of Oradea, 410087 Oradea, Romania

**Keywords:** BGP-15, telemetry, arrhythmia, heart rate variability, isoproterenol, echocardiography

## Abstract

Multi-target drug candidate BGP-15 has shown cardioprotective and antiarrhythmic actions in diseased models. Here, we investigated the effects of BGP-15 on ECG and echocardiographic parameters, heart rate variability (HRV), and arrhythmia incidence in telemetry-implanted rats, under beta-adrenergic stimulation by isoproterenol (ISO). In total, 40 rats were implanted with radiotelemetry transmitters. First, dose escalation studies (40–160 mg/kg BGP-15), ECG parameters, and 24 h HRV parameters were assessed. After, rats were divided into Control, Control+BGP-15, ISO, and ISO+BGP-15 subgroups for 2 weeks. ECG recordings were obtained from conscious rats, arrhythmias and HRV parameters were assessed, and echocardiography was carried out. ISO-BGP-15 interaction was also evaluated on an isolated canine cardiomyocyte model. BGP-15 had no observable effects on the ECG waveforms; however, it decreased heart rate. HRV monitoring showed that BGP-15 increased RMSSD, SD1, and HF% parameters. BGP-15 failed to counteract 1 mg/kg ISO-induced tachycardia, but diminished the ECG of ischemia and suppressed ventricular arrhythmia incidence. Under echocardiography, after low-dose ISO injection, BGP-15 administration lowered HR and atrial velocities, and increased end-diastolic volume and ventricle relaxation, but did not counteract the positive inotropic effects of ISO. Two weeks of BGP-15 treatment also improved diastolic function in ISO-treated rats. In isolated cardiomyocytes, BGP-15 prevented 100 nM ISO-induced aftercontractions. Here, we show that BGP-15 increases vagally mediated HRV, reduces arrhythmogenesis, enhances left ventricle relaxation, and suppresses the aftercontractions of cardiomyocytes. As the drug is well tolerated, it may have a clinical value in preventing fatal arrhythmias.

## 1. Introduction

The incidence of sudden cardiac death (SCD) due to lethal arrhythmias is increasing, despite the slight decline in cardiovascular deaths [1]. A considerable proportion of cardiovascular disease-related deaths are the direct or indirect result of cardiac arrhythmias [1]. Ventricular arrhythmias cause death in around fifty percent of individuals with chronic heart failure (HF) [2]. Compared to certain other therapeutic areas, cardiovascular drug research has had relatively little investment during the previous years. In addition, many antiarrhythmic drug candidates failed to show a clear benefit in reducing mortality; therefore, there is still a need for new antiarrhythmic treatments. [3,4]. In the last decade, it became apparent that the activity of the autonomic nervous system (ANS) and cardiovascular mortality, especially SCD, have a strong association. The idea of measuring heart rate variability (HRV) as a quantitative indicator of autonomic activity has been inspired by experimental findings that link the susceptibility to fatal arrhythmias either to increased sympathetic or decreased vagal tone [5,6]. Low HRV is associated with an increased risk of SCD and all-cause mortality in post-myocardial infarction sufferers and HF patients [7,8]. According to several studies, low vagally mediated HRV has been postulated as a predictive marker of increased mortality and susceptibility to fatal ventricular arrhythmias in post-myocardial infarction and HF patients [9,10]. Subsequently, beta-adrenergic overstimulation (i.e., by isoproterenol) is a useful tool to dose-dependently generate stress-induced cardiomyopathy, heart failure, and arrhythmias in rodent models and is also used for arrhythmia induction in human patients as a diagnostic aid in basic electrophysiology studies [11,12]. Murine models have been widely used in arrhythmia research and translational studies aimed to evaluate the effects of drugs on HRV. However, as anesthetics alter the autonomic regulation of the heart, radiotelemetry is the only acceptable method to assess HRV in conscious, unrestrained experimental animals, which makes these experiments more complicated and expensive [5,13]. Nonetheless, to date, various computer-based radiotelemetry systems are available on the market to assess short- and long-term HRV in different animal models.

Substantial evidence suggests that BGP-15 drug candidate (3-piperidino-2-hydroxy-1-propyl)-nicotinic amidoxime) exerts various cardioprotective actions and can improve cardiac function in animal models of heart disease, although its main mode of action has remained elusive [14,15]. It has been shown that BGP-15-treatment reduces the incidence of atrial fibrillation (AF) episodes in a transgenic mouse model of HF, and this effect has been associated with the increased phosphorylation of the cardiac-specific insulin-like growth factor 1 (IGF1) receptor [16]. Additionally, BGP-15 acts as an inhibitor of poly(ADP)-ribose polymerase 1 (PARP-1) [17]. According to a recent study, PAPR-1 inhibition protects against contractile dysfunction and atrial fibrillation, preserving cardiomyocyte function [18]. It has been described that BGP-15 protects against mitochondrial ROS production and oxidative damage by modulating PARP-1 in ischemia-reperfusion injury performed in the Langendorff heart perfusion system [19]. Several reports suggest that BGP-15 modulates plasma membrane composition through lipid-raft reorganization and increases membrane fluidity, which is crucial because stiff membranes are limiting the cellular stress response [20]. Furthermore, it has been demonstrated that BGP-15 has a beneficial effect against hypertension-induced cardiac remodeling and cardiac fibrosis by decreasing the activity of transforming growth factor beta (TGFβ/Smad) and mitogen-activated protein kinase (MAPK) signaling. It favorably influences the prosurvival signaling pathways and activates mitochondrial biogenesis, thereby resulting in an increase in mitochondrial mass [21]. In addition, our team recently showed that BGP-15 decreases cardiovascular mortality, recovers disrupted mitochondrial homeostasis, and increases mitochondrial ATP production in obese rats [22]. It has been recently stated that developing agents that diminish the mitochondrial ROS overload has enormous potential in preventing lethal ventricular arrhythmias [23]. Based on the above-described effects of BGP-15 on mitochondria, the drug candidate may play an essential role in reducing arrhythmogenesis. This assumption is further reinforced by the study, which reports that the drug candidate reportedly stabilizes Kir2.1 current in long-QT7 channelopathy [24]. Based on previous results, the drug candidate BGP-15 delays the onset of diastolic dysfunction and elevates the level of phosphorylation of VASP and phospholamban in Goto-Kakizaki rats with diabetic cardiomyopathy [25]. As phospholamban is a key regulator of cardiac contractility and modulates SR Ca^2+^ sequestration by inhibiting the sarco-endoplasmic reticulum Ca-ATPase (SERCA) in its dephosphorylated state, BGP-15 may act by restoring SERCA activity [26,27]. Our team has previously shown that BGP-15 enhances cyclic guanosine monophosphate (cGMP)—protein kinase G (PKG) signaling, alters myocardial mechanics, and furthermore, decreases inotropy in paced human right atrial samples [28,29].

Based on the above, we investigated the effects of BGP-15 on the electrocardiogram (ECG), isoproterenol-induced cardiac arrhythmias, heart rate variability, and echocardiographic heart functions in a telemetry-implanted rat model. Here, we show that BGP-15 reduces arrhythmogenesis, increases vagally mediated HRV, improves diastolic function, and suppresses the aftercontractions of cardiomyocytes. As the substance is proven to be well-tolerated in phase II human studies [30], we propose to further evaluate its cardioprotective actions in patients suffering heart failure and cardiac arrhythmias.

## 2. Results

### 2.1. Effects of BGP-15 Dose-Escalation on Heart Rate and ECG Parameters of Naïve Rats

**Figure 1:**
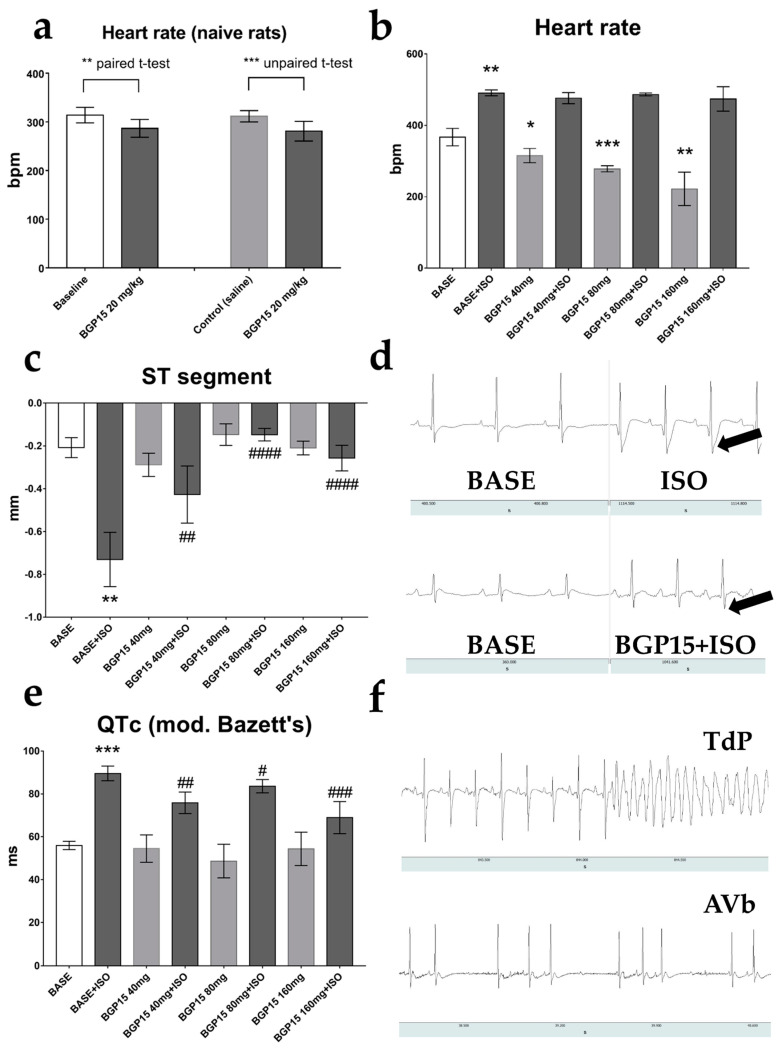
Effects of BGP-15 dose-escalation on ECG parameters of telemetry-implanted rats. (**a**) BGP-15 (20 mg/kg) decreased mean heart rate. (**b**) BGP-15 treatment (40–160 mg) decreased heart rate but failed to prevent the isoproterenol-induced tachycardia. (**c**) ISO-induced ST segment depression was counteracted by all BGP-15 doses. (**d**) Representative ECG recording of unrestrained rats under baseline conditions (left panels), and under the influence of isoproterenol (1 mg/kg, upper right) and BGP-15 (40 mg/kg, bottom right) + isoproterenol (1 mg/kg). Arrows indicate depressed ST segments. (**e**) The effects of ISO and BGP-15 on corrected QT intervals (Bazett’s formula, modified for rats). QT-prolongation was observed after the wear-off of isoproterenol-induced tachycardia, which was counteracted by BGP-15 treatment. (**f**) Representative telemetric recordings. Upper panel: 2 mg isoproterenol-induced torsade de pointes (after, exitus); bottom panel: co-administration of 2 mg/kg ISO and 160 mg/kg BGP-15 caused AV block. Normal distribution was assessed by the Shapiro–Wilk normality test. Asterisks (*) denote the level of significance compared to BASE (*: *p* < 0.05; **: *p* < 0.01; ***: *p* < 0.001; Student’s *t*-test), and hash symbols (#) denote significance compared to BASE+ISO (#: *p* < 0.05; ##: *p* < 0.01; ###: *p* < 0.001; ####: *p* < 0.0001; Student’s *t*-test). Abbreviations: bpm: beats per minute; ISO: isoproterenol; TdP: torsade de pointes; AVb: atrioventricular block.

Oral administration of 20 mg/kg, 40 mg/kg, 80 mg/kg, and 160 mg/kg BPG-15 slightly and dose-dependently reduced the heart rate of conscious rats. BGP-15 (20 mg/kg, the average dose based on previous studies) lowered HR compared to the Control (saline-treated) rats (*p* = 0.0037, unpaired *t*-test), and also compared to corresponding baseline values of the animals (*p* = 0.005, paired *t*-test; Figure 1a). BGP-15 administration (40–160 mg/kg) failed to acutely counteract 1 mg/kg ISO-induced tachycardia (ISO was injected i.p. 10 min after BGP-15 administration; Figure 1b). No significant effects on ECG waves were attainable on the lead-II electrocardiogram of naïve BGP-15-treated rats (data not shown).

### 2.2. Effects of BGP-15 Dose-Escalation on ECG Parameters of ISO-Treated Rats

Although oral BGP-15 administration did not alter normal lead-II ECG waveforms, 40–160 mg/kg BGP-15 pre-treatment significantly counteracted ischemic signs (depression of the ST-segment) caused by 1 mg/kg ISO (*p* = 0.029, BASE vs. BASE+ISO; and *p* = 0.0062, BGP40 vs. BASE+ISO; *p* < 0.0001, BGP80 vs. BASE+ISO; and *p* < 0.0001, BGP160 vs. BASE+ISO, respectively; Figure 1c,d). A significant prolongation of the QTc interval (modified Bazett’s formula) was shown on the lead-II ECG of 1 mg/kg ISO-treated rats after the wear-off of the drug-induced tachycardia (*p* = 0.0003, BASE vs. BASE+ISO), which was again counteracted by 40–160 mg/kg BGP-15 administration (Figure 1e). In our preliminary studies, higher doses of ISO (2 mg/kg and 5 mg/kg) caused significant mortality by triggering torsade de pointes-type lethal arrhythmias, recorded by the telemetry system (Figure 1f, upper panel). We note that in one ISO-treated animal, overdosing with BGP-15 (160 mg/kg BGP-15) caused 2nd-degree AV block (Wenkebach type; Figure 1f, bottom panel).

### 2.3. Effects of Single-Dose BGP-15 on 24-h HRV Parameters

BGP-15 treatment (40 mg/kg) had an observable effect on 24 h HRV parameters of telemetry-implanted rats (paired *t*-test, Table 1). The average HR was lower during the treatment (*p* = 0.0497, BASE vs. BGP-15). In the time domain measurements, the standard deviation of R-R intervals (SDNN) and the proportion of the successive NNs that differed by more than 10 ms (pNN10) tended to increase. The root mean square of the successive differences (RMSSD, indicative of parasympathetic activity) increased during the BGP-15 treatment (*p* = 0.0294, BASE vs. BGP-15). In nonlinear analyses (Poincaré plot), we found that SD1 (the parameter correlated to RMSSD) increased significantly (*p* = 0.0270, BASE vs. BGP-15). In frequency-domain measurements, there was no significant change in the VLF band; however, the LF band % decreased, and the HF band % (another index of vagal activity) significantly increased (*p* = 0.0016, BASE vs. BGP-15).

### 2.4. Ehocardiographic Results of the Acute Treatment Groups

**Figure 2:**
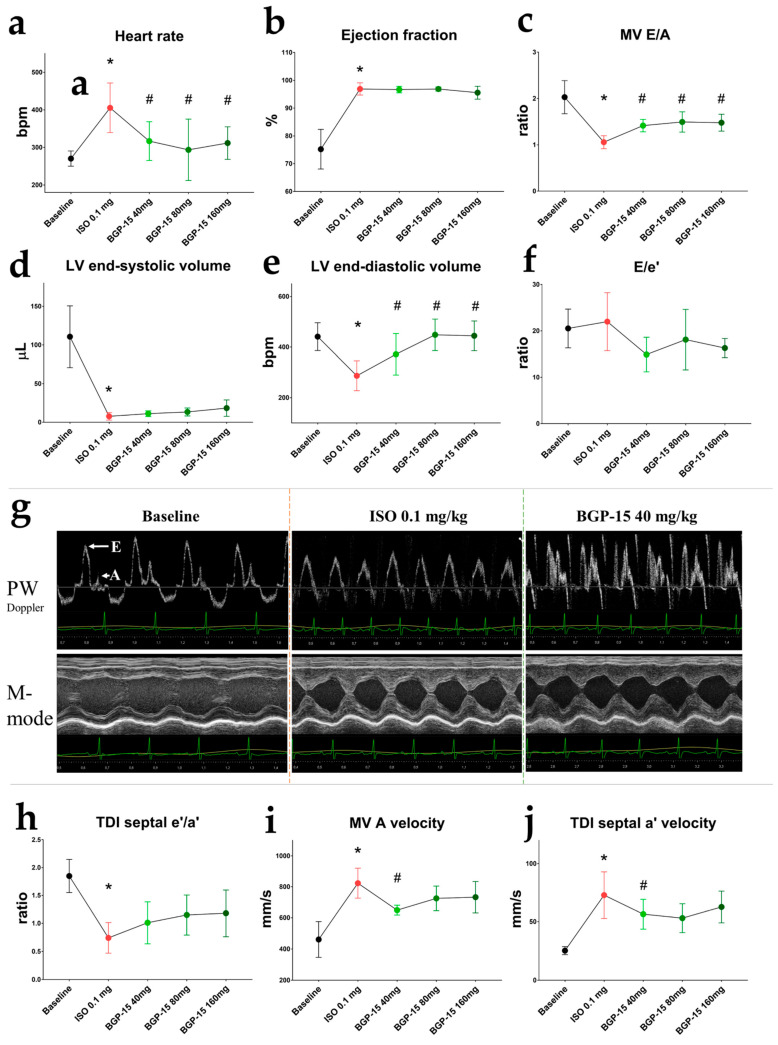
Effects of 40 mg/kg, 80 mg/kg, and 160 mg/kg cumulative doses of BGP-15 on echocardiographic parameters of ISO-treated rats. (**a**) HR increased after ISO administration but decreased after all doses of BGP-15. (**b**) BGP-15 failed to counteract the positive effect of ISO on LV ejection fraction. (**c**) Transmittal E/A decreased after ISO but increased under the influence of BGP-15. (**d**) Left ventricle end-systolic volume decreased after ISO but tended to increase after BGP-15 injections. (**e**) Left ventricle end-diastolic volume increased after BGP-15 dosing. (**f**) Changes in E/e′ ratios (indicative for LV filling pressure) were only tendentious. (**g**) Representative echocardiographic traces of treatment groups. Upper panels: transmitral PW Doppler images; bottom panels: M-mode traces of the LV (PSLAX view). (**h**) Tissue Doppler e′/a′ ratio decreased after ISO injection. (**i**) Transmitral atrial wave (A) velocity decreased after BGP-15 administration (**j**) Septal atrial (a′) velocity decreased under the influence of BGP-15. Normal distribution was assessed by the Shapiro–Wilk normality test. Asterisks (*) denote significance compared to the Baseline group, and hashmarks (#) denote significance compared to the ISO 0.1 mg group (*p* < 0.05, paired Student’s *t*-test).

Echocardiography was performed using a high-resolution ultrasound machine under ketamine/xylazine (50/5 mg/kg) anesthesia. Baseline traces were recorded 15 min after the injection of the anesthetics, after ensuring that the heart rate was under 300 bpm (HR: 270.3 ± 20.31 bpm) to properly assess diastolic waves. Isoproterenol administration (0.1 mg/kg, i.p.) elevated HR (405.5 ± 65.97 bpm; *p* = 0.0025 vs. baseline; Figure 2a). All tested doses of BGP-15 (40 mg/kg, 80 mg/kg, and 160 mg/kg cumulative doses, i.p.) decreased ISO-induced tachycardia (with p-values: *p* < 0.0001, *p* = 0.0003, and *p* = 0.0018 vs. ISO 0.1, respectively). Ejection fraction (EF) dramatically increased after ISO administration (*p* = 0.0006, reaching 96.9 ± 2.186%; Figure 2b). All doses of BGP-15 increased LV end-diastolic volume (*p* = 0.0115, *p* = 0.0021, and *p* = 0.0064 vs. ISO 0.1 mg, respectively; Figure 2e), and a trend toward increase was seen in LV end-systolic volume (Figure 2d). Despite this, BGP-15 injections failed to significantly counteract the positive effects of ISO on EF. Diastolic function was evaluated by pulsed-wave Doppler and tissue velocity imaging (TDI). The transmitral E/A ratio decreased after ISO dosing (E and A wave fusion was seen in some cases), but recovered after BGP-15 administration (*p* = 0.0054, BGP-15 40mg/kg vs. ISO 0.1 mg; Figure 2c), as the drug decreased A wave velocity (*p* = 0.0024, BGP-15 40mg/kg vs. ISO 0.1 mg; Figure 2i). Similar results were obtained from tissue Doppler measurements: the TDI e′/a′ ratio slightly increased (Figure 2h), and septal a′ wave velocity significantly decreased after BGP-15 injections (Figure 2j). The ratio of E/e′ (indicative of LV filling pressure) tended to decrease after 40 mg/kg BGP-15 administration (Figure 2f,g).

### 2.5. Effects of 2-Week ISO and BGP-15-Treatments on 2-h HRV Parameters

**Figure 3:**
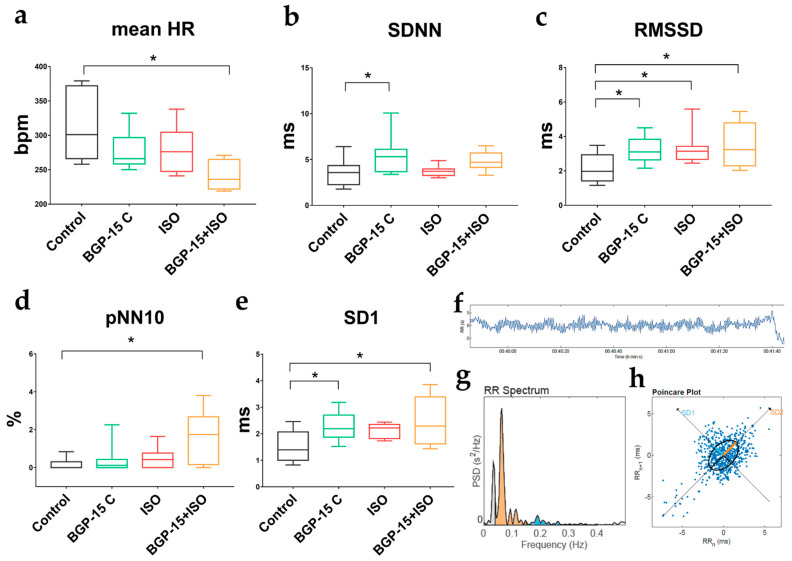
Effects of 2-week BGP-15 and ISO treatment on resting HRV parameters of conscious rats. (**a**) Two-week-long administration of BGP-15 (40 mg/kg) slightly, but BGP-15+ISO significantly decreased mean HR (in resting conditions, after wear-off of acute ISO-induced tachycardia). (**b**) BGP-15 treatment increased total variability (described by SDNN: standard deviation of the NN (R-R) intervals). (**c**) RMSSD (root mean square of the successive differences, indicative of increased parasympathetic tone) increased in all treated groups. (**d**) pNN10% (proportion of the number of pairs of successive NNs that differ by more than 10 ms) increased significantly in the BGP-15+ISO group. (**e**) SD1 (standard deviation of the width of the Poincaré plot ellipse, indicative of short-term variability in the parasympathetic tone) significantly elevated in the BGP-15-treated groups of rats. (**f**) Representative images of HRV analyses: filtered tachogram. (**g**) the R-R spectrum (Fast Fourier Transformation) showing VLF (grey), LF (orange), and HF (blue) bands. (**h**) representative non-linear analysis (Poincaré plot). Normal distribution was assessed by the Shapiro–Wilk normality test. Asterisks (*) denote significance between groups (if *p* < 0.05). One-way ANOVA, Tukey’s post-test.

Short-term HRV parameters were evaluated on the last day of the 2-week-long treatments, at resting conditions (after the wear-off of the ISO-induced tachycardia). Interestingly, the most significant decrease in mean HR was observed in the BGP-15+ISO group (Figure 3a; *p* = 0.0022 vs. control) along with the highest increase in pNN10 (Figure 3d; *p* = 0.0007 vs. control). Total variability (defined as SDNN, Figure 3b) increased in the BGP-15 treated rats (*p* = 0.0084 vs. control). RMSSD and SD1 (indices of vagal activity) increased in the BGP-15-treated (*p* = 0.036 and *p* = 0.018, respectively) and BGP-15+ISO-treated animals (*p* = 0.0064 and *p* = 0.0022, respectively) compared to controls (Figure 3c,e).

### 2.6. Effects of the 2-Week-Long ISO and BGP-15 Treatments on Echocardiographic Parameters

Endpoint echocardiographic parameters were evaluated on the last day of 2-week long treatments (Table 2). No significant differences were found in LV dimensions and systolic parameters (EF, CO) between the groups. Left ventricle mass increased in the ISO group (*p* = 0.0147 vs. control). BGP-15 treatment did not counteract this effect of ISO. However, more distinct changes were found in diastolic parameters. ISO treatment lengthened deceleration time (*p* = 0.0291 vs. control) and tended to decrease the e′/a′ ratio (*p* = 0.09 vs. control). The ratio of e′/a′ improved in the BGP-15+ISO group (*p* = 0.0318 vs. ISO), moreover, BGP-15 treatment increased TDI s’ velocity (*p* < 0.0001 vs. control). Finally, the ratio of E/e′ (indicative of LV filling pressure) improved in both the BGP-15-treated group compared to the control (*p* = 0.0041), and in the ISO+BGP-15 group compared to ISO (*p* = 0.0307).

### 2.7. Effects of BGP-15 on ISO-Induced Arrhythmogenesis

**Figure 4:**
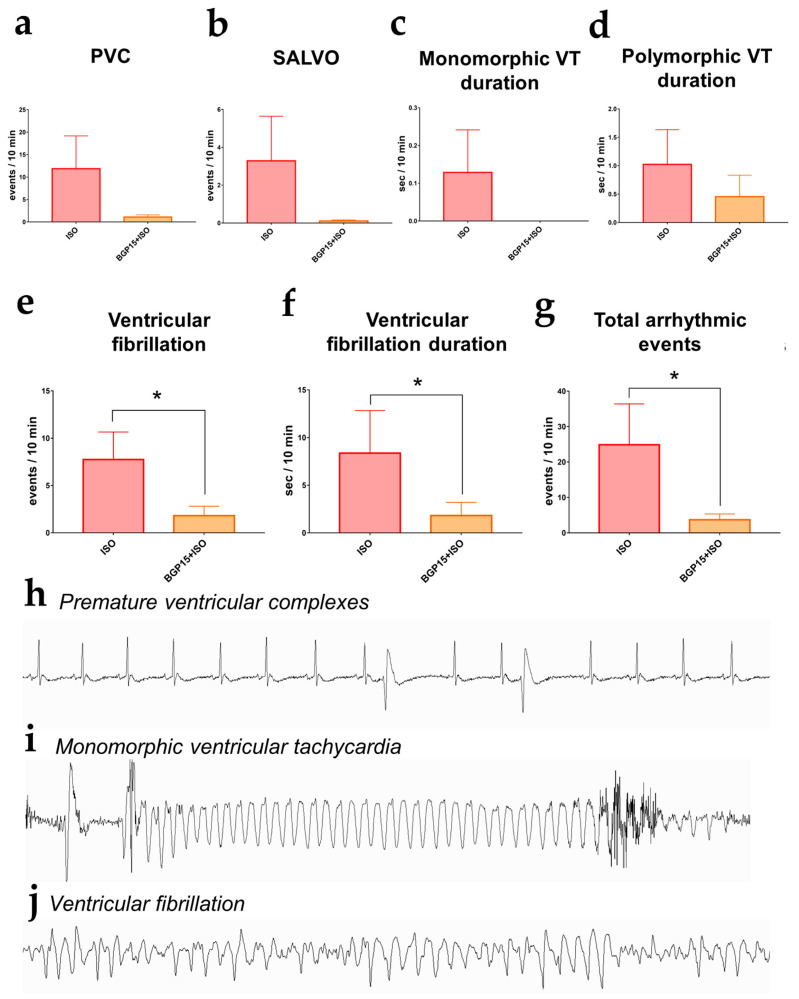
Arrhythmic events (interpolated to a 10 min period) recorded on the last day of Protocol II. (**a**) Number of premature ventricular complexes (PVCs). (**b**) The incidence of salvos. (**c**) Duration of monomorphic ventricular tachycardia. (**d**) Duration of polymorphic ventricular tachycardia. (**e**) The number of ventricular fibrillation events significantly decreased in the BGP-15-treated rats. (**f**) The duration of ventricular fibrillation was shorter in the BGP-15+ISO group. (**g**) The total number of arrhythmic events was decreased in the BGP-15-treated animals. Data distribution was assessed by the Shapiro–Wilk normality test. Groups were compared by the Mann–Whitney test; *: *p* < 0.05. (**h**) Telemetric lead II ECG recording of ventricular premature complexes. (**i**) ECG recording of a monomorphic ventricular tachycardia (**j**) ECG trace of a ventricular fibrillation episode (all representative recordings were obtained from the experiments).

The ventricular arrhythmic events were evaluated on 10 min long telemetric Lead II ECG recordings on the last day of the 2-week-long ISO and BGP-15+ISO treatments (n = 17). We counted the number of premature ventricular complexes (*PVC*s), salvos, duration (s) of monomorphic and polymorphic ventricular tachycardia (VT), ventricular fibrillation (VF), and the duration (s) of VF episodes. The total number of arrhythmic events was significantly lower in the BGP-15+ISO group compared to ISO (*p* = 0.045; Figure 4g). *PVCs* and VT duration tended to decrease in the BGP-15-treated rats. (Figure 4a–d). The number of VF episodes was significantly decreased in BGP-15+ISO vs. ISO (*p* = 0.0308; Figure 4e), along with the duration of VF episodes (*p* = 0.0185; Figure 4f).

### 2.8. Results of Isolated Cardiomyocyte Experiments

**Figure 5:**
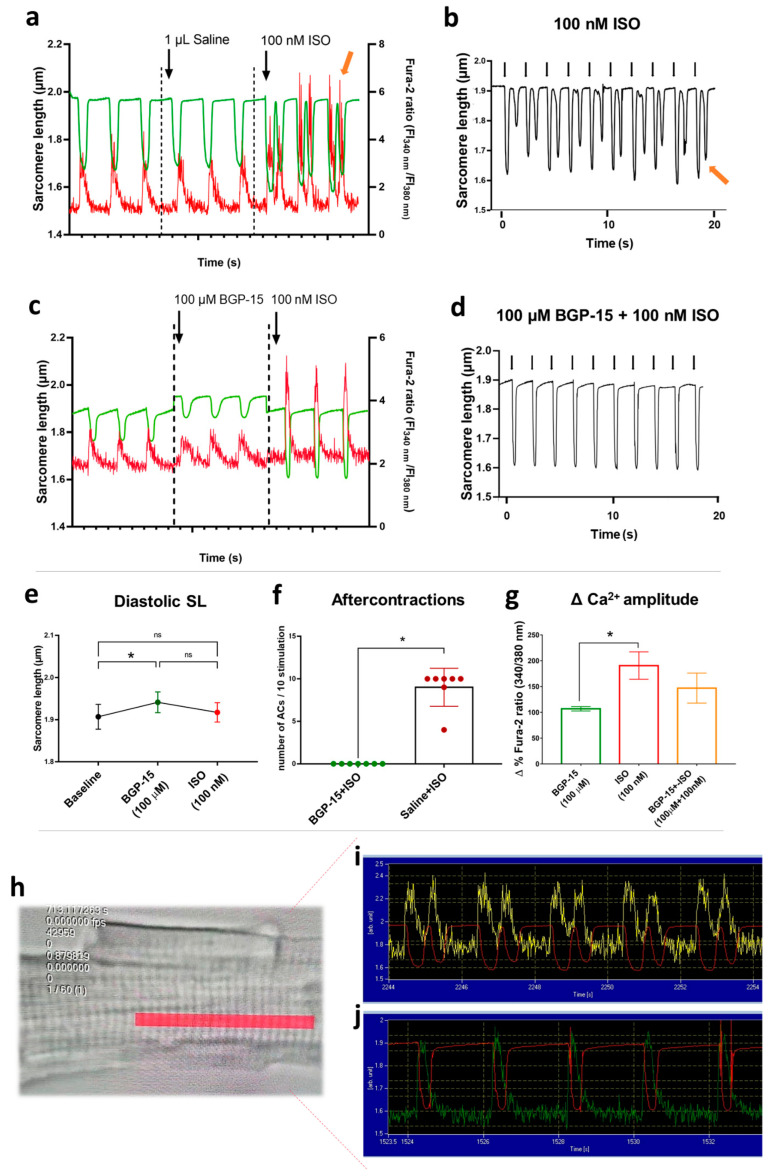
Effects of BGP-15 and ISO treatment in isolated cardiomyocytes. (**a**) After vehicle pre-treatment, the application of 100 nM ISO evoked aftercontractions and increased Ca^2+^-transient amplitudes (green line shows the sarcomere length, red line indicates Ca^2+^-transients, and the yellow arrow shows the 3rd aftercontraction with a spontaneous Ca^2+^ peak). (**b**) Representative magnified image illustrates sarcomere length changes during ISO treatment (black arrows for electrical stimulation, yellow arrow for the last aftercontraction). (**c**) Pre-treatment with 100 µM BGP-15 increased diastolic sarcomere length and prevented ISO-induced aftercontractions. (**d**) Magnified image for sarcomere length shows the combined effects of BGP-15 and ISO treatments (black arrows for electrical stimulation). Note the absence of aftercontractions. (**e**) 100 µM BGP-15 increased diastolic sarcomere length, but ISO treatment blunted this effect. (**f**) The number of ISO-induced aftercontractions (ACs) during 10 field stimulations in BGP-15 or saline pre-treated cardiomyocytes. Data distribution was assessed by the Shapiro–Wilk normality test. Groups were compared by the Mann–Whitney test, *p* < 0.05. (**g**) Magnitude of i.c. calcium peak amplitude (in the percentage of the corresponding baseline values) in Fura-2-loaded cardiomyocytes. BGP-15 alone did not affect Ca^2+^ transients, but ISO application roughly doubled the amplitude. BGP-15 pre-treatment partly counteracted the effects of ISO on Ca^2+^ transient amplitude. One-way ANOVA, Tukey’s post-test (*: *p* < 0.05). (**h**) Representative image of an isolated cardiomyocyte. The red area shows the region of interest, selected for the sarcomere length—Ca transient measurements. (**i**) Raw data represents the Ca transients (yellow line) and the sarcomere length (red line) in an ISO-treated cardiomyocyte. Note the aftercontractions. (**j**) Raw data represents the Ca transients (green line) and the sarcomere length (red line) in an ISO-treated cardiomyocyte in the presence of BGP-15.

Changes in sarcomere length and Ca^2+^-transient amplitude were evaluated in single, isolated canine cardiomyocytes at a stimulation frequency of 0.5 Hz. In the presence of physiologic saline solution, 100 nM ISO elicited aftercontractions (in almost all cardiomyocytes following ISO applications; Figure 5a,b,f) and increased the amplitudes of Ca^2+^-transients to 190.6 ± 65.16% from baseline values (100 ± 40.43%) (Figure 5g). Pre-treatment with 100 µM BGP-15 prevented ISO-induced aftercontractions in all cardiomyocytes tested (Mann–Whitney test, *p* = 0.0006; Figure 5c,d,f), although it did not counteract the effect of ISO on Ca^2+^-transient amplitude (Figure 5g) (*p* = ns, BGP-15 vs. BGP-15+ISO). Interestingly, BGP-15 treatment increased the resting (diastolic) sarcomere length (*p* = 0.0342, Figure 5e) of the isolated cardiomyocytes.

## 3. Discussion

Here, we demonstrate that the small molecule BGP-15 reduces arrhythmogenesis, prevents ECG changes resulting from beta-adrenergic overstimulation, increases vagally mediated HRV, and improves diastolic function in telemetry-implanted rats. At the cellular level, we showed that BGP-15 suppresses isoproterenol-induced aftercontractions and the reactivation of Ca^2+^ transients in isolated cardiomyocytes.

Previous human studies have shown that BGP-15 is well tolerated and does not exert any side effects on cardiac electrophysiology [30]. Our results corroborate these findings. As a result of BGP-15 administration to telemetry-implanted, conscious rats, BGP-15 did not have any visible effects on resting lead II ECG waves; however, it slightly (but significantly) decreased heart rate. In contrast, BGP-15 application failed to counteract 1 mg/kg ISO-induced tachycardia; thus, we conclude that BGP-15 possesses no (or very weak) antagonistic effects on beta-adrenergic receptor-mediated positive chronotropy, despite showing some similarity to propranolol [28]. ISO administration causes severe tachycardia with transient ischemia, which is apparent on the ECG (T-wave inversion, ST segment changes [31,32,33]. Accordingly, in our studies, ST-segment depression was seen on the ECG recordings under the influence of ISO (we note that it is difficult to detect the ST segment in the rat ECG as it is very short and the T wave often rises in continuity with the S wave) [34]. BGP-15 pre-treatment counteracted ISO-induced ST-segment changes, while unaffected acute tachycardia. In addition, after the wear-off of the ISO effect, significant QT-interval prolongation was detected on the resting ECGs, possibly due to the resulting myocardial ischemia that affects repolarization [35]. This was also prevented by BGP-15 pre-treatment, as the resting QTc interval was normalized. We were also able to record one torsade de pointes type fatal arrhythmia during the preliminary experiments (Figure 1f). We note that in rats treated with high dose (2 mg/kg) ISO and BGP-15 (160 mg/kg), AV blocks were seen after BGP-15 application, which might be the result of BGP-15 overdose on injured hearts.

In the following series of experiments, we evaluated the effects of BGP-15 (40 mg/kg) on heart rate variability (HRV). HRV itself describes the fluctuation in the interval between subsequent heartbeats and is generated by heart–brain interactions and dynamic autonomic nervous system activities. An optimal level of HRV is linked to good health, self-regulation, adaptation, and resilience [36,37]. Elevated sympathetic activity, accompanied by age-related deterioration in cardiac vagal regulation, impairs the electrical stability of both the atria and the ventricles and triggers arrhythmogenesis [38,39]. Low vagally mediated HRV has been postulated as a predictive marker of increased mortality and susceptibility to fatal ventricular arrhythmias [9,10]. In contrast, interventions (various drugs, exercise training, and vagal nerve stimulation) that increase vagally mediated HRV are proposed to exert significant cardioprotection and decrease the risk of SCD [40,41,42]. We found that over a 24 h period, BGP-15 treatment decreased the resting mean heart rate in undisturbed rats, while it did not affect total variability (SDNN), but increased RMSSD. Since feedbacks driven by the vagal nerve operate quickly, the RMSSD represents high-frequency oscillations of R-R intervals and therefore estimates vagal activity [37]. According to the non-linear analyses, we found that BGP-15 also increased the SD1 value (indicative of short-term parasympathetic (PS) variability). Spectral analysis of 24 h HRV data revealed similar findings as BGP-15 increased the relative power of the high-frequency band (HF%), another significant measure of PS tone [43]. Thus, we conclude that BGP-15 treatment increases vagally mediated HRV. These findings also corroborate previous results showing some similarity in the cardiac actions of BGP-15 and beta-blockers (propranolol) [28]. Of note, in the beta-blocker heart attack trial, propranolol treatment also increased the recovery of PS tone in patients with acute myocardial infarction as measured by HF power and RMSSD, thus, improving survival [44]. We also obtained short-term HRV data on rats treated with vehicle, BGP-15, ISO, or BGP-15+ISO after a 2-week treatment period. In these studies, similar findings were obtained, as BGP-15 treatment slightly increased total variability (SDNN) and significantly elevated indices of vagal tone (RMSSD and SD1). The resting heart rate of the ISO group was normal, but variability was slightly increased, in accordance with literature data [45]. We conclude that BGP-15 increases vagally mediated HRV, which may contribute to arrhythmia prevention.

Echocardiographic changes were also evaluated after single-bolus and 2-week-long BGP-15 and ISO treatments, with congruent results. ISO significantly elevated HR and dramatically increased LV ejection fraction (LVEF). However, because of the severe tachycardia, E and A waves were fused, and end-diastolic volume and e′/a′ ratio decreased. BGP-15 lowered HR (we note that animals were under ketamine-xylazine anesthesia in this case), and also counteracted tachycardia induced by a minimal dose (0.1 mg/kg) of ISO, without any significant effect on LVEF. Despite this, BGP-15 administration (40 mg/kg i.p.) increased LV end-diastolic volume, E/A and e′/a′ ratios, indicative of enhanced diastolic function. We note that, as BGP-15 injection lowered HR, the increased EDV might be a consequence of the increased filling time. The increase in the E/A ratio might also be a result of the decreased heart rate; however, BGP-15 also decreased TDI a′ wave velocity, a parameter relatively independent of HR [46]. Transmitral A wave was also decreased after the treatment, corresponding to our previous findings that BGP-15 has negative tropic effects on the human atria [28]. Similar outcomes were obtained from the 2-week studies, as BGP-15 treatment did not affect systolic parameters (EF, CO), but improved diastolic function, corresponding to our previous results in different animal models [25,29]. In addition, in the 2-week studies, we found worsened diastolic function (E/A, DecT) and significantly elevated LV mass in the ISO group, in accordance with the literature data [47]. Notably, we found that BGP-15 treatment significantly counteracted these effects of ISO. To summarize, we conclude that BGP-15 improves diastolic function and exerts cardioprotective actions against constant beta-adrenergic damage.

The main result of this telemetric study is that BGP-15 administration is able to prevent various types of ventricular arrhythmias resulting from adrenergic stimulation. We found that the total number of arrhythmic events was significantly lower in the BGP-15+ISO group compared to ISO. The number and duration of VF episodes significantly decreased, and the incidence of premature complexes and ventricular tachycardia also tended to decline. To our knowledge, this is the first study showing that BGP-15 may have value in preventing ventricular arrhythmias. Nevertheless, Sapra and colleagues demonstrated the antiarrhythmic actions of BGP-15 in two different mouse models of heart failure and atrial fibrillation (HF+AF), but they focused only on the supraventricular events (AF) [16]. Here, we show that BGP-15 may also prevent ventricular arrhythmic episodes resulting from adrenergic stimulation.

To elicit experimental Ca^2+^-mediated afterdepolarizations in cardiac cells, ISO is widely used (100 nM-1µM), as it causes Ca^2+^ overload [48,49]. High-dose isoproterenol causes alterations in action potential dynamics in cardiomyocytes, causing either delayed or early afterdepolarizations (DAD or EAD, respectively). Beta-adrenergic EAD shares a common ionic mechanism with DAD in terms of cellular Ca^2+^ overload and spontaneous sarcoplasmic reticulum (SR) Ca^2+^ release [50,51,52]. ISO elevates PKA-dependent phosphorylation of L-type calcium channels (LTCC) and ryanodine (RyR2) receptors, which results in enhanced Ca^2+^ signals [53]. Thus, EADs and DADs induced by beta-adrenergic stimulation mediate arrhythmogenesis in the intact and diseased myocardium, which is associated with calcium overload, impaired contractility, and increased arrhythmia incidence [54]. Here, we showed aftercontractions as a result of re-activated Ca^2+^-transients in all cardiomyocytes after 100 nM ISO treatment. (We note that as we did not directly measure the action potentials but the sarcomere length of the cells, the term ‘aftercontraction’, instead of ‘afterdepolarization’, is more appropriate, as suggested by Egdell [55]). The amplitude of the Ca^2+^ signal was significantly increased compared to baseline conditions, which corroborates to literature data [49,56]. BGP-15 pre-treatment (100 µM) before ISO application completely prevented aftercontractions of isolated cells, which might be the cellular basis of its antiarrhythmic properties. The Ca^2+^-transients measured in the presence of BGP-15 tended to decrease when compared to ISO controls, showing that BGP-15 may partly affect Ca^2+^ overload in cardiomyocytes. Interestingly, BGP-15 caused an increase in diastolic (and resting) sarcomere length, which might be a result of its actions on titin phosphorylation, which was previously shown by our team [29].

Considering these results, we propose some possible mechanisms by which BGP-15 prevents arrhythmogenesis. Firstly, we have recently shown that BGP-15 increases cardiac cGMP-PKG signaling [29]. PKG exerts cardioprotection by improving the diastolic function [57], but also modulates ATP-sensitive potassium channels (K(ATP) channels) via the inhibition of the (Ca^2+^/calmodulin-dependent protein kinase II (CaMKII) pathway, a major contributor to arrhythmogenesis [58]. K(ATP) activation reduces Ca^2+^ oscillations and cell contractility, thus stabilizing energy consumption [58]. The reduced Ca^2+^ peaks and aftercontractions observed in the BGP-15 pre-treated cells might be the result of the above processes, however, this hypothesis needs further evaluation. The cGMP-dependent signals activate SERCA via PKG. The increase in SERCA activity appears to diminish Ca^2+^ peaks and Ca^2+^ oscillations and attenuate hypercontracture [59]. This effect of BGP-15 on the SERCA pump was also shown by our team; thus, we suggest that this mechanism also contributes to suppressing aftercontractions [29]. PKG activation also takes part in mitochondrial mitoK(ATP) channel regulation, transmitting cardioprotective signals, and restoring mitochondrial respiration [60]. Substantial evidence suggests mitochondrial protective actions of BGP-15, while the disrupted mitochondrial Ca^2+^ homeostasis is a major contributor to arrhythmogenesis [21,23,25,61]. Mitochondrial ATP ensures that SR Ca^2+^-handling proteins operate properly, providing that mitochondria receive an appropriate supply of Ca^2+^ ions. Myocytes from HF models showed an increased mitochondrial Ca^2+^ peak, AP prolongation, EADs QT prolongation, and a high incidence of ventricular arrhythmia following the application of ISO [62]. Moreover, genetic knockdown of mitochondrial Ca^2+^ uniporters inhibited mitochondrial Ca^2+^ uptake, reduced Na^+^-Ca^2+^ exchange currents, and suppressed EADs, reducing ventricular fibrillation [63]. Thus, the mitoprotective actions of BGP-15 may further contribute to suppressing aftercontractions and arrhythmias [61].

Another possible explanation is that BGP-15 reportedly stabilizes the Kir2.1 current in Andersen–Tawil syndrome (long-QT7) channelopathy, thus protecting the electrical stability of the cardiac cells [24]. Lastly, on the organism level, we demonstrated that BGP-15 administration increases vagally mediated HRV, thus increasing the adaptation capacity of the heart while decreasing its vulnerability to arrhythmias [64].

There are several limitations to this current report. For instance, testing multiple doses of isoproterenol might have provided a better insight into the potential influence of BGP-15 on isoproterenol-induced tachycardia. Furthermore, animal models themselves have various limits on assuming translational perspectives in preclinical drug research. Here, we used a rat model which may resemble the human heart less closely than larger mammals, e.g., rabbits or swine models. Last, further studies are needed to clarify the exact molecular mechanism of action of BGP-15.

To summarize the above, substantial evidence supports the cardioprotective abilities of BGP-15. As this drug candidate is proven to be well tolerated, we suggest further evaluating its clinical value in patients suffering from heart failure and cardiac arrhythmias.

## 4. Materials and Methods

### 4.1. Animal Model and Chemicals

All experimental protocols were approved by the local Ethics Committee of the University of Debrecen (12/2022 DEMÁB, August 12, 2022), and the animals received humane care in accordance with the “Principles of Laboratory Animal Care” by EU Directive (citation). Animal experiments are reported in compliance with the ARRIVE guidelines [65]. In total, 40 male adult Sprague-Dawley rats (493.77 ± 17.56 g, Charles River Laboratories Inc., Germany) were housed in a room with controlled temperature and kept under a 12/12 h dark/light cycle. A 2-week adaptation period was provided before the initiation of the treatments. BGP-15 (Sigma-Aldrich-Merck KGaA, Darmstadt, Germany) was dissolved freshly in saline before oral gavage or intravenous (i.v.) bolus administration (depending on the study protocol). Myocardial injury was induced by isoproterenol (Sigma-Aldrich-Merck KGaA, Darmstadt, Germany), dissolved freshly in saline. For antibiotic prophylaxis and postoperative care, cefuroxime-sodium (50 mg/kg) was used intraperitoneally. Metamizole-sodium (100 mg/kg) was administered on the first 5 postoperative days as analgesic therapy.

### 4.2. Study Design

In Protocol I (dose escalation and single-dose treatments), isoproterenol (ISO) was administered intraperitoneally (i.p.) to telemetry implanted rats (n = 8, in 0.1, 1, 2, and 5 mg/kg cumulative doses), and ECGs were recorded during the 15 min preceding, and 3 h after the injections. BGP-15 (20, 40, 80, and 160 mg/kg) was orally administered to another population of implanted rats, and an ECG was recorded. After a wash-off period (1 week), BGP-15 dose escalation was performed, which was followed by the injection of ISO (1 or 2 mg/kg, i.p) to evaluate the interactions of the two drugs on heart rate and ECG parameters. Finally, another population of rats was subjected to 0.1 mg/kg i.p. ISO treatment and then BGP-15 dose escalation (40, 80, and 160 mg/kg, i.p.) under echocardiographic monitoring (ketamine/xylazine: 50/5 mg/kg anesthesia, continuous ECG recording). Data was evaluated, and the ISO (1 mg/kg) and BGP-15 (40 mg/kg) doses were chosen to perform the long-term studies. The 1 mg/kg ISO dose, according to our ECG findings and literature data [66,67], is sufficient to produce arrhythmias, transient myocardial ischemia, and injury.

In Protocol II (2-week follow-up), rats (n = 32) underwent telemetry implantation, and a 10-day long recovery period was provided. Following a 24-h ECG recording, a single oral BGP-15 bolus (40 mg/kg) was administered, followed by another 24-hour ECG recording of conscious animals to assess the effects of BGP-15 on HRV parameters. After, rats were divided into 4 groups: Control (vehicle-treated), BGP-15 (40 mg/kg), ISO (1 mg/kg), and ISO+BGP-15 (1 mg/kg and 40 mg/kg, respectively). ISO was administered i.p. on the 1st, 4th, 8th, and 12th days. ECG recordings were performed all day for 2 h, and, on the days of the ISO treatment, during the 15 min that preceded and the 3 h that followed the injections. All injections were performed between 10.00 and 12.00 h. On the 14th day, echocardiography was performed, and rats were sacrificed. On the lead II ECG recordings of the 13th day, short-term HRV parameters were determined, and arrhythmic events were counted. In addition, isolated canine cardiomyocyte experiments were carried out to evaluate BGP-15—ISO interactions (see Section 4.7).

### 4.3. Surgical Implantation of Radiotelemetry Transmitters

Aseptic conditions were assured by using autoclaved instruments and sterilized materials and by disinfecting the workbench. Following antiseptic processes, the telemetry transmitters (Stellar Implantable Transmitter, Type PBTA-L1; Stellar Telemetry, TSE Systems Inc., Chesterfield, MO, USA) were first prepared. After being removed from their sterile package, the leads of the transmitters were shortened to a length appropriate for the size of the animals, considering their growth during research as well. Telemetry device implantations were performed under ketamine-xylazine anesthesia (100/15 mg/kg, i.m.). After the loss of the righting reflex, hair clipping was performed on both the thoracic and interscapular regions of the animals. Transmitter implantation into the animal’s back is considered to be less invasive than intraperitoneal implantation, ensuring a higher survival rate and fewer movement-based artifacts on the ECG [13]. Next, three skin incisions were made: a mid-clavicular longitudinal incision (about 1 cm in length) located on the right side of the thorax at the level of the 2nd and 3rd ribs approximately; a 2 cm long transverse incision located on the left side around the level of the 7th rib; and a transverse incision ≤5 cm on the dorsal skin in the interscapular region. The areas of the wounds were s.c. prepared, as well as the route from the skin incisions on the chest to the dorsal incision. Following this, the telemetry devices (ECG sampling frequency: 1000 Hz) were placed under the dorsal skin into the pouch formerly created by preparing the subcutaneous connective tissue. From here, the electrodes were subcutaneously transferred to the chest through the incisions and positioned according to the 2nd ECG chest lead. Real-time ECG recording was used to find the optimal placement of the electrodes. After fixing the electrodes in the right position with holding stitches (polypropylene, 5/0), disinfection was performed, and wounds were closed with continuous horizontal mattress sutures (4/0, polyglactin 910). A heated table was used to maintain body heat during and after the surgery. Post-operative care consisted of the administration of antibiotics (cefuroxime 50 mg/kg, i.p.) and analgesic medications (metamizole-sodium 50 mg/kg, i.m.), observation, wound care, and weight measurements for 3 consecutive days after surgery in individually housed rats. Animals were provided with a 10-day long recovery period before the initiation of the treatments.

### 4.4. ECG Monitoring and Detection of Arrhythmias

ECG (sampling frequency: 1000 Hz) and locomotor activity (LOC) were recorded from undisturbed rats in their cages. Radio signals were picked up by a receiver (Stellar Receiver and Antenna, Stellar Telemetry, TSE Systems Inc., Chesterfield, MO, USA) and recorded by a computer instrumented with the AcqKnowledge ACK100-STL Biopac Software (BIOPAC Systems Inc., Goleta, CA, USA). Signals were recorded for 35 s in every 2 min for 2 h (short-time HRV protocol) or 5 min every hour for a day (24 h protocol). The frequency of spontaneous and drug-induced cardiac arrhythmias was determined and quantified offline. The incidence of premature ventricular complexes (PVC), salvos, monomorphic and polymorphic ventricular tachycardia duration, ventricular fibrillation and its duration, and the total number of arrhythmic events (reported as the number of events per 10 min) were identified and quantified, according to the most recent guideline (The Lambeth Conventions II) [68].

### 4.5. Heart Rate Variability (HRV) Analyses

From the continuous ECG data, R-R interval raw data (tachogram) were generated for each 2 min recording period. ECG R waves were automatically identified and exported by the AcqKnowledge ACK100-STL Biopac Software (BIOPAC Systems Inc., CA, USA). Each raw ECG tachogram was first visually examined to make sure that all R-waves had been appropriately identified. ECG recording segments that showed artifacts (the animal was moving, the signal was noisy, or an arrhythmic event occurred) were eliminated without replacement and were excluded from further investigation. A filter was applied to ensure the exclusion of abnormally low or high R-R intervals (100–300 ms). Heart rate (as beats per minute, bpm) and HRV parameters were quantified using the KUBIOS HRV software (version 3.4.2., Kubios Inc., Finland) with modifications in accordance with Thireau and colleagues’ criteria for assessing HRV parameters in rodents [13]. Subsequent R-R differences greater than 30 ms were also excluded by the software, and the remaining arrhythmias were automatically filtered again. The normal (N) R-R intervals were considered N-N intervals. Time-domain measures included the standard deviation of the time between normal-to-normal beats (SDNN), the root mean square of successive beat-to-beat interval differences (RMSSD), and the proportion of the number of pairs of successive NNs that differ by more than 10 ms (pNN10%) [13]. For frequency-domain HRV data (only for the 24 h HRV experiments), a power spectrum was generated by a fast Fourier transform-based method (Welch’s periodogram: 256 points, 50% overlap, and Hamming window), automatically by the KUBIOS software. The powers (ms2) of the very-low-frequency (VLF: <0.04 Hz), low-frequency (LF: 0.04–0.78 Hz), and high-frequency (HF: 0.78–2.5 Hz) bands were calculated [43]. Non-linear analyses included the automatic generation of a Poincaré plot and the determination of the SD1 parameter (standard deviation of the width of the Poincare plot ellipse, indicative of short-term variability). Each tachogram was split into 2 min epochs (0–2 min, 2–4 min, etc.), and, for each epoch, separate indices of HRV and ΔHR values were generated. Subsequently, parameters were averaged as the mean values of animals [42]. To assess the effects of BGP-15 and ISO, HR and HRV values were calculated for baseline conditions (pre-injection) and after the wear-off of the ISO-induced tachycardia, at resting conditions, in two-hour long ECG signals. HR and HRV data were analyzed by means of paired *t*-tests (Protocol I, to estimate differences between two groups) or one-way ANOVA, Tukey’s post-test (Protocol II, to estimate differences between 4 groups).

### 4.6. Echocardiography

In both acute and long-term studies, echocardiography was carried out with the Vevo 3100 ultrasound imaging system (Fujifilm VisualSonics Inc., Toronto, ON, Canada) equipped with a high-frequency transducer (MX250, 14–28 MHz), designed for cardiac imaging of rodents. The imaging was performed under ketamine-xylazine anesthesia (50/5 mg/kg, i.m.) after chest hair removal and placing the animals onto a heated table (Visualsonics SR200, adjusted to 39 ± 0.5 °C). Data acquisition was performed in accordance with the recommendations of the American Society of Echocardiography [69]. The database was built from the parasternal long- and short-axis, as well as from the apical four-chamber view (PSLAX, PSAX, and A4C, respectively). The aortic arch was visualized from a modified suprasternal view. After acquisition, data were stored and offline analyzed by a blinded reader using the VevoLAB software (version 5.1, Fujifilm VisualSonics Inc., Toronto, ON, Canada).

Left ventricle (LV) internal diameter in end-diastole (LVIDd, mm) and end-systole (LVIDs, mm), anterior, and posterior LV wall thickness (mm, LVAWd, LVAWs, and LVPWd, LVPWs, respectively) were determined by manually tracing the endo-and epicardial borders in M-mode traces. Left ventricular volume in end-diastole and end-systole (LVVOLd, LVVOLs, respectively; µL) was calculated by the software. LV ejection fraction (LV EF, %) was calculated as 100*(LVVOLd-LVVOLs)/LVVOLd. Left atrial (LA) size (mm) and aortic (Ao) diameter (mm) were measured from M-mode recordings. Heart rate (HR, bpm), stroke volume (SV, µL), cardiac output (CO, ml/min), and corrected LV mass (mg) were determined (1.053*((LVIDd+LVPWd+IVSd)3-LVIDd3)*0.8).

Diastolic function was evaluated by pulsed-wave Doppler (PW) and tissue Doppler imaging (TDI) from apical 4-chamber views. PW Doppler echocardiography was used to measure the ratio of the peak early (E) and atrial (A) transmitral flow velocities (E/A ratio) and the E wave deceleration time (DecT, ms). Wall motion velocity was defined as the e′/a′ ratio by measuring e′ and a′ waves at the septal annulus by TDI. The E/e′ ratio (indicative of LV filling pressure) was also calculated. Aortic flow velocities (Vel, peak and mean, mm/s) and pressure gradients (PGrad, peak and mean, mmHg) were measured after visualizing the aortic arch from a modified suprasternal view. Three cardiac cycles were averaged for each parameter; data are presented as the mean ± SD.

### 4.7. Isolated Canine Cardiomyocyte Experiments

Canine cardiomyocytes were donated by the Department of Physiology, University of Debrecen. The cells were isolated from the midmyocardial region, as described elsewhere [70]. Briefly, hearts were isolated from anesthetized (ketamine: 10 mg/kg, xylazine: 1 mg/kg) adult beagle dogs and single cardiomyocytes were obtained by enzymatic dispersion technique using Joklik solution (Minimum Essential Medium Eagle, Joklik Modification; Sigma-Aldrich Co., St. Louis, MO, USA) supplemented with type II collagenase (1 mg/mL) for 35 min. Isolated cells were kept at 15 °C and were subjected to ex vivo calcium transient measurements. Cardiomyocytes were loaded with 5 μM Fura-2 AM for 30 min in Tyrode solution, in the presence of Pluronic F-127. Cardiomyocytes were then allowed to rest for 30 min to let the intracellular esterases cleave Fura-2 AM, then stored at 15 °C until the measurements. Cells were then placed in a tissue bath on the stage of an inverted microscope (Nikon Eclipse TS100, Nikon Corp., Tokyo, Japan). The final volume of the chamber filled with Tyrode solution (containing 144 mM NaCl, 5.6 mM KCl, 2.5 mM CaCl_2_, 1.2 mM MgCl_2_, 5 mM HEPES, and 11 mM dextrose, at pH = 7.4) was 1 mL. After sedimentation, a single rod-shaped cardiomyocyte with a clear striation pattern was chosen to record the physiological parameters. The cardiomyocyte was paced (Experimetria, MDE, Heidelberg) by 0.5 Hz. Alternating excitation wavelengths of 340 and 380 nm were used to monitor the fluorescence signals of Ca^2+^-bound and Ca^2+^-free Fura-2 dye, respectively. Fluorescent emission was detected at 510 nm, and traces were digitized at 200 Hz using the FeliX software (Ratiomaster RM-50 system, Horiba, New Brunswick, NJ, USA). The experimental protocol was the following: after a 5 min resting period, cardiomyocytes were paced at 0.5 Hz for 30 secs, resulting in a steady-state condition. The baseline resting sarcomere length was determined after a 1 min stabilization period following the pacing. Then, 1 µL BGP-15 (100 µM, dissolved in distilled water) or 1 µL saline (control) was added to the chamber, followed by 5 min of incubation. Sarcomere length was determined again, and a 0.5 Hz pacing was initiated for at least 30 s. Finally, 1 µL isoproterenol (ISO, dissolved in distilled water) was added at a final concentration of 100 nM, and pacing was initiated for another 30 s cycle with the recording of the above parameters. Background fluorescence intensity was obtained at the end of the measurements in a region without cells and was subtracted from the original data. Sarcomere length and Ca^2+^-transient amplitude of cardiomyocytes were exported to GraphPad Prism software, and the curves were analyzed with appropriate curve-fitting methods.

### 4.8. Statistics and Data Analysis

Data are presented as the mean value of the group ± standard error of the mean (SEM), unless stated otherwise. Group numbers were determined based on the minimum numbers required for statistical analyses, also considering our previous studies and possible losses due to the treatments. Experiments were designed to generate groups of equal size using randomization and blinded analysis. Statistical analyses were carried out only for experiments where each group size was at least n = 5, both in animal and cellular studies. First, the Gaussian distribution of data was estimated by the Shapiro–Wilk normality test. Statistical analysis was then performed using one-way analysis of variance (ANOVA), followed by Tukey’s multiple comparisons post-test (only if F in the ANOVA achieved *p* < 0.05, the normality test was passed, and there was no significant variance inhomogeneity) or Kruskal–Wallis test followed by Dunn’s post-test (when the normality test was not passed). Student’s *t*-test or Mann–Whitney test (non-Gaussian data) was used to estimate differences between two treatment groups (unpaired or paired, depending on the control/self-control design). Statistical analyses were carried out using GraphPad Prism software for Windows, version 8.00 (GraphPad Software Inc., La Jolla, CA, USA). Outliers were identified as mean ± 2SD when it was necessary. Probability values (p) less than 0.05 were considered significantly different and marked with an asterisk (*: *p* < 0.05, relative to controls) or a hash symbol (#: *p* < 0.05, relative to ISO). Group sizes represent the number of independent samples/animals, not technical replicates.

## Figures and Tables

**Table 1:** Twenty-four hour HRV parameters of telemetry-implanted rats in baseline conditions (BASE) and during 40 mg/kg BGP-15 treatment. Normal distribution was assessed by the Shapiro–Wilk normality test. Asterisks denote significance (*p* < 0.05, paired Student’s *t*-test).

Parameter	BASE 24 h	40 mg/kg BGP-15 24 h	Significance
Heart rate (bpm)	372 ± 32	322 ± 28	*
SDNN (ms)	2.185 ± 0.5572	2.627 ± 0.5348	ns
RMSSD (ms)	1.819 ± 0.7211	2.485 ± 0.6293	*
pNN10 (ms)	0.1055 ± 0.1698	0.5351 ± 0.4455	ns
SD1 (ms)	1.274 ± 0.5263	1.761 ± 0.4447	*
SD2 (ms)	2.785 ± 0.6812	3.205 ± 0.7143	ns
LF %	23.09 ± 24.48	12.62 ± 5.244	ns
HF %	27.95 ± 14.9	54.32 ± 13.41	*
VLF %	38.78 ± 11.9	39.54 ± 10.56	ns

bpm: beats per minute; SDNN: standard deviation of the NN (R-R) intervals; RMSSD: root mean square of the successive differences; pNN10: the proportion of the number of pairs of successive NNs that differ by more than 10 ms divided by the total number of NNs; SD1: standard deviation of the width of the Poincare plot ellipse; SD2: standard deviation of the length of the Poincare plot ellipse; LF: low frequency; HF: high frequency, VLF: very low frequency.

**Table 2:** Endpoint echocardiographic parameters of rats (Protocol II). Data distribution was assessed by the Shapiro–Wilk normality test. Asterisks (*) denote significance to control; and hash symbols (#) shows significance to ISO (*p* < 0.05, one-way ANOVA, Tukey post-test).

Parameter	Control	BGP-15	ISO	ISO+BGP-15
LA/Ao ratio	1.164 ± 0.37	0.814 ± 0.244	0.645 ± 0.112	0.887 ± 0.315
Heart Rate (bpm)	258 ± 29	254 ± 17	250 ± 30	252 ± 40
LV Vol d (µL)	372.4 ± 79.28	352.5 ± 40.81	329.7 ± 100.2	362.2 ± 77.27
LV Vol s (µL)	98.54 ± 47.0	73.28 ± 24.13	77.15 ± 54.61	75.81 ± 29.86
SV (µL)	273.8 ± 45.07	279.2 ± 28.06	252.5 ± 52.36	286.3 ± 63.03
EF (%)	77.61 ± 7.51	79.47 ± 5.348	76.38 ± 11.02	79.32 ± 7.121
CO (ml/min)	70.69 ± 13.94	70.88 ± 9.278	63.49 ± 16.94	71.44 ± 16.07
LV mass corr. (mg)	1089 ± 189.5	1262 ± 193.9	1385 ± 266.5 *	1335 ± 205.4 *
E/A ratio	1.841 ± 0.273	2.046 ± 0.39	1.907 ± 0.377	1.907 ± 0.331
DecT (ms)	42.59 ± 12.18	44.91 ± 14.77	61.5 ± 18.71 *	47.69 ± 12.59
TDI s’ (mm/s)	43.3 ± 13.17	65.68 ± 10.14 *	52.39 ± 9.155	57.32 ± 6.569 *
TDI e′ (mm/s)	36.53 ± 10.62	44.07 ± 7.595	33.4 ± 9.435	44.74 ± 9.092
TDI a′ (mm/s)	32.06 ± 10.49	43.51 ± 18.13	41.22 ± 11.49	34.95 ± 8.726
e′/a′ ratio	1.205 ± 0.305	1.116 ± 0.5303	0.7586 ± 0.357	1.338 ± 0.244 #
E/e′ ratio	26.44 ± 6.565	18.55 ± 3.237 *	25.66 ± 4.967	18.14 ± 4.88 *,#

LA: left atrium; Ao: aorta; bpm: beats per minute; LV: left ventricle; Vol: volume; d: diastolic; s: systolic; SV: stroke volume; EF: ejection fraction; CO: cardiac output; E/A: ratio of transmitral early and atrial peak inflow; DecT: deceleration time of the early wave; TDI: tissue Doppler imaging: systolic tissue velocity at the septal annulus; e: early diastolic velocity at the septal annulus; a: atrial diastolic velocity at the mitral annulus; E/e′: ratio of early transmitral and early tissue velocities.

## Data Availability

The data that support the findings of this study are available from the corresponding author upon reasonable request. Some data may not be made available because of privacy or ethical restriction.

## Abbreviations

AF: atrial fibrillation; ANS: autonomic nervous system, cGMP: cyclic guanosine monophosphate; ECG: electrocardiogram; EF: ejection fraction; HF: heart failure; HRV: heart rate variability; hsp: heat-shock protein; ISO: isoproterenol; LF: low-frequency; LV: left ventricle; PARP-1: poly(adenosine 5-diphosphate)–ribose]polymerase 1; PKA: protein kinase A; PKG: protein kinase G; PLB: phospholamban; pNN10: proportion of the successive NNs that differ by more than 10 ms; PS: parasympathetic; PVC: premature ventricular complex; RMSSD: Root mean square of the successive differences; ROS: reactive oxygen species; SCD: sudden cardiac death; SDNN: standard deviation of R-R intervals; SERCA2a: sarco/endoplasmic reticulum Ca^2+^-ATPase; VLF: very low frequency.
